# From Exome to Optical Mapping: Resolving a Complex 17q12 Rearrangement Underlying a Severe Neurodevelopmental Disorder

**DOI:** 10.21203/rs.3.rs-11055592/v1

**Published:** 2026-10-09

**Authors:** Zain Dardas, Haowei Du, Daniel G. Calame, Davut Pehlivan, Elif Yilmaz Gulec, Ahmed K. Saad, P. J. Hastings, Jennifer E. Posey, Richard A. Gibbs, Christopher M. Grochowski, James R. Lupski

**Affiliations:** Baylor College of Medicine; Baylor College of Medicine; Baylor College of Medicine; Baylor College of Medicine; Istanbul Medeniyet University; Baylor College of Medicine; Baylor College of Medicine; Baylor College of Medicine; Baylor College of Medicine; Baylor College of Medicine; Baylor College of Medicine

**Keywords:** Complex genomic rearrangement (CGR), Neurodevelopmental disorder (NDD), Optical genome mapping (OGM), Parental mosaicism

## Abstract

**Background:**

Complex genomic rearrangements (CGRs) are significant contributors to neurodevelopmental disorders, yet their full structural architecture often eludes conventional diagnostic methods. We investigated the genetic basis of a 7-year-old boy presenting with severe intellectual and growth delay, immune deficiency, and multiple congenital anomalies.

**Methods:**

We combined exome sequencing, array comparative genomic hybridization, short-read and long-read genome sequencing, droplet digital PCR, and optical genome mapping to characterize a chromosomal rearrangement identified in the proband and to assess segregation within the family trio.

**Results:**

This integrated approach reconstructed the architecture of a highly complex duplication-triplication-quadruplication rearrangement on chromosome 17q12, encompassing the developmental and immune-regulatory genes *DUSP14*, *SYNRG*, *DDX52*, and *HNF1B*, consistent with the proband’s phenotypic spectrum. Breakpoint mapping revealed microhomologies and inverted repeats, implicating a replication-based mechanism, such as microhomology-mediated break-induced replication, in the rearrangement’s formation. Droplet digital PCR further uncovered low-level mosaicism in the asymptomatic father, indicating a potential germline transmission pathway relevant to familial recurrence risk.

**Conclusions:**

Integrating multiple sequencing and optical mapping technologies can resolve complex structural variation, uncover parental mosaicism, and implicate candidate genes that conventional methods alone would miss. This approach improves molecular diagnosis and advances understanding of the mutational mechanisms underlying complex disease-associated rearrangements.

## Background

Complex genomic rearrangements (CGRs) represent a critical yet underappreciated class of structural variants (SVs) with significant implications for human disease. CGRs are defined as structural alterations in the genome that involve more than one breakpoint junction and often include a combination of copy number variants (CNVs), inversions, and translocations^1,2^. These rearrangements can be extensive, involving large segments of the genome, and their full resolution and clinical interpretation pose significant challenges.

Recent studies have highlighted the substantial contribution of CGRs to rare diseases and rare disease traits (as defined in OMIM; http://www.omim.com), including neurodevelopmental disorders (NDDs)^3–5^. The increased use of next-generation sequencing (NGS) technologies in both clinical and research settings has uncovered the prevalence and complexity of these genomic events. CGRs are particularly relevant in the context of NDDs, where they can disrupt critical genes and regulatory regions, leading to a range of phenotypic outcomes. The impact of such disruptions is substantial given that approximately 1/3 of all 22,000 computationally annotated genes in the human genome are expressed in the nervous system^6^, making the brain uniquely vulnerable to genomic instability.

Identifying CGRs requires a multifaceted approach, combining various genomic technologies to resolve the complexities of these variant alleles. Traditional cell and cytological based methods, such as karyotyping and fluorescence *in situ* hybridization (FISH), offer a broad overview of chromosomal aberrations but lack the resolution needed to detect finer details. Chromosomal microarray (CMA) provides more detailed insights into CNVs but cannot detect balanced chromosomal rearrangements (BCRs) like inversions and translocations. Advancements in genome sequencing (GS), particularly with the advent of short-read (> 75bases/read; sr-GS) and long-read (lr-GS) sequencing technologies, have significantly improved our ability to detect and characterize CGRs. These technologies allow for the phasing of multiple breakpoint junctions and the resolution of SVs within repetitive regions, which are often the sites of genomic instability. The integration of GS with other methodologies, such as optical mapping and array comparative genomic hybridization (aCGH), has proven effective in revealing the complex architecture of CGRs^7^.

Neurodevelopmental disorders (NDD), characterized by impairments in cognitive, behavioral, and motor functions, often have a genetic basis that includes single-nucleotide variants (SNVs), indels, as well as CGRs. For example, deletions or duplications of specific genomic regions can result in dosage imbalances of critical genes, while inversions and translocations can alter gene regulation and expression.

For instance, a patient with a CGR involving multiple inversions on chromosome 6 exhibited features of Coffin–Siris syndrome, highlighting how such rearrangements can disrupt essential developmental genes like *ARID1B*^8^. Similarly, complex rearrangements involving the *MECP2* gene have been associated with *MECP2* duplication syndrome, a severe neurodevelopmental disorder^9^.

In this study, we employed a multi-tiered genomic strategy to resolve a CGR in a previously unsolved severe neurodevelopmental syndrome case. Our investigation revealed parental mosaicism and identified genes, *DDX52* and *DUSP14*, that might play a role in contributing to the observed phenotype. This comprehensive approach underscores the value of integrating various genomic data types to unravel the genetic architecture of NDD, ultimately contributing to more accurate diagnoses and improved patient care.

## Methods

### Patient Enrollment

The family was enrolled under a protocol approved by the institutional review board at Baylor College of Medicine (IRB #: H-29697) as part of the Turkish brain malformation cohort^10^. Genomic DNA was extracted from peripheral blood using standard protocols.

### Exome Sequencing and Analysis

Exome sequencing (ES) was conducted at the Baylor College of Medicine Human Genome Sequencing Center using established protocols^11^. The process began with whole-exome capture employing the Human Genome Sequencing Center’s core design (52 Mb, Roche NimbleGen, RRID: nif-0000–31466) and continued with sequencing on the HiSeq platform (Illumina) achieving approximately 150× depth of coverage. The sequence data were then aligned and mapped to the human reference genome (hg19) using the Mercury in-house bioinformatics pipeline. Variant calling was performed with the ATLAS (an integrative variant analysis pipeline optimized for variant discovery) and SAMTOOLS (RRID: nlx_154607, the Sequence Alignment/Map) suites, and annotations were conducted using a custom annotation pipeline. This pipeline leverages genetic variant annotations along with additional tools and databases. To detect copy number variants (CNVs) from exome sequencing data, including duplications and deletions not readily identified by standard variant callers, we applied HMZDupFinder^12^, a computational tool that infers copy number changes from normalized exome read-depth data relative to an in-house control cohort.

### Array Comparative Genomic Hybridization (aCGH)

Initial aCGH analyses were performed using a 60K custom designed Agilent array (AMADID#: 086718) that spans multiple regions including 500 kb in 17q12. A subsequent custom 180K Agilent high-resolution array was designed to interrogate the long arm of chromosome 17 (AMADID#: 086829). Array experiments were conducted following manufacturer protocols for hybridization and labeling with minor modifications^13^.

### Validation and Segregation analysis of identified CNVs with ddPCR

Identified CNVs were validated and assessed for segregation across the trio using droplet digital PCR (ddPCR) on the QX200^™^ AutoDG^™^ Droplet Digital^™^ PCR System (Bio-Rad), run according to standard manufacturer guidelines. Prior to amplification, genomic DNA samples were digested with either RsaI or AluI (New England Biolabs) to disrupt linkage that could otherwise mask tandem duplication signals. Each reaction was prepared in a 20 μl volume consisting of 10 μl 2x ddPCR Supermix for Probes (no dUTP), 0.25 μl of each primer (10 μM stock), 250 nM probe, and 50 ng of digested genomic DNA. A FAM-labeled hydrolysis probes were used to target the regions of interest within the CGR, paired with a HEX-labeled reference probe/primer set targeting *RPP30*, consistent with prior methodology^12^. Following automated droplet generation, samples underwent thermal cycling under the following conditions: an initial 95°C step for 5 minutes; 40 cycles of 95°C (30 s), 60°C (1 min), and 72°C (1 min); a 90°C step for 5 minutes; and a final indefinite hold at 4°C, with all transitions set to a 2°C/s ramp rate. Droplets were then read and analyzed in QuantaSoft^™^ Software (Bio-Rad). Copy number calls were derived from the relative concentration of FAM- and HEX-positive droplets (expressed as positive droplets per μl) for each sample^14^

### Short-Read Genome Sequencing (sr-GS)

Genome sequencing (GS) was conducted on DNA derived from the peripheral blood leukocytes of the proband and parents at the Human Genome Sequencing Center (HGSC) at Baylor College of Medicine. The library preparation utilized a PCR-free protocol with a 550-bp insert size, facilitated by the KAPA Hyper Prep kit. Sequencing was performed on the Illumina NovaSeq 6000 platform, producing 150 bp paired-end reads. The sequencing data typically meet quality control metrics including an average genome coverage of over 40X, with more than 97.5% of target bases (digital exome) covered at over 20X. Data analysis and interpretation were carried out using the Baylor Genetics analytics pipeline. The raw sequencing data, initially in BCL format, were converted to FastQ files based on each sample’s adapter sequence using Illumina’s recommended procedures. These FastQ files were then aligned to the human reference genome build GRCh38 using the Illumina Dragen BioIT Platform, resulting in BAM files. Quality control (QC) metrics for the alignment process, such as coverage of digital exome regions, matepair alignment information, and counts of total and duplicate reads, were recorded for quality review. Integrative genomic viewer (IGV) was utilized to visualize the sequencing data for the trio and identify the breakpoint junction sequences.

### Long-Read Genome Sequencing (lr-GS)

Pacific Biosciences (PacBio) lr-GS was carried out on the trio at the HGSC at Baylor College of Medicine. Initially, DNA quality was assessed using Qubit and pulsed-field gel electrophoresis (PFGE). The sequencing library was then prepared using 5 μg of genomic DNA with the SMRTbell Express Template Preparation Kit, aiming for an average fragment length of 15 kb. This library was then sequenced across five SMRTcells on the PacBio Sequel II system, generating an average of 43 Gb of HiFi reads per sample and a coverage of 15–20x.

### Optical Genomic Mapping (OGM)

Ultra-high molecular weight (UHMW) DNA was extracted from frozen EDTA blood according to the manufacturer’s guidelines. Initially, the frozen samples were thawed at 37°C. The blood samples were then quantified using a HemoCue WBC System (HemoCue AB). Subsequently, a volume containing 1.5 million cells was centrifuged at 2,200x g for 5 minutes to form pellets. These pellets were resuspended in a DNA stabilization buffer and subjected to proteinase K treatment in lysis and binding buffers. Following proteinase K digestion, the samples were treated with phenylmethylsulfonyl fluoride (PMSF), bound to a nanobind disk, washed, and then eluted. The DNA extracts were then homogenized by end-over-end rotation and incubated at room temperature overnight prior to fluorescent labeling. For fluorescent labeling, 750 ng of DNA per sample was labeled at the CTTAAG recognition site using Direct Labeling Enzyme 1 (DLE-1) as per the manufacturer’s protocol. The labeled DNA was analyzed on a Saphyr Gen2 platform, achieving 400X-1500X effective coverage per dataset. *De novo* assembly and structural variant calling were conducted using Bionano Solve version 3.7, following the guidelines outlined in the Bionano Solve Theory of Operation: Structural Variant Calling. To optimize the computational time for *de novo* assemblies, each dataset was reduced to 250X effective coverage by selecting the longest molecules. Structural variants were identified relative to the human reference genomes hg19 and hg38. Using the Variant Annotation Pipeline, these variants were annotated and compared against the Bionano control sample database, which contains over 600,000 SV calls from more than 150 phenotypically normal individuals across over 26 populations. The raw molecules in each contig present in the OGM data were analyzed to detect molecules that encapsulated both breakpoint junctions in cis, enabling the interpretation of sequence motifs in each genomic fragment to elucidate haplotype inferences and differences within individuals.

## Results

### Clinical Presentation

The proband, BAB10751, a 7-year-old boy, presented with a complex clinical profile characterized by global developmental delay and growth retardation, immune deficiency, and distinct facial features including hypotelorism, upslanting palpebral fissures, a flat depressed nasal bridge, and a wide prominent philtrum (Fig. 1). Additionally, he has multiple congenital anomalies including esophageal atresia, tracheoesophageal fistula, duodenal atresia, and short bowel syndrome, with part of his bowel removed due to ischemia. Renal issues were also significant, with nephrolithiasis, renal cysts, and secondary renal failure noted. His stature is notably short, possibly aligning with his overall growth delay. His parents were related, and there was no family history of disease.

### Genomic Rearrangement Architecture and Breakpoint Junction Sequences

Initial exome sequencing analysis for SNVs and Indels did not resolve the genetic basis of the proband’s condition. A subsequent reanalysis using HMZDupFinder, which utilized normalized exome read count data, identified a ~ 232kb duplication when compared with a control group in our inhouse database (Fig. 2A). This finding prompted further investigation via array CGH, which confirmed a complex genomic rearrangement (CGR) comprising an apparent duplication-triplication (DUP-TRP) event (Fig. 2B). This rearrangement spans regions involving multiple brain-expressed genes—*DUSP14, SYNRG, DDX52*, and *HNF1B*—that could potentially be linked to NDDs. The terminus of the CNV falls within a highly repetitive region on chromosome 17, which was not adequately covered by a high-resolution array spanning the predicted CNV region. Therefore, we designed another array that spans the entire 17q12 and used it to test the trio DNA. However, the distal end of the CNV could not be resolved due to the presence of inverted repeats in this region (Supplementary Fig. 1).

Array CGH for the trio revealed a minor increase in copy number in the father, suggesting the possibility of mosaicism in this genomic region (Fig. 2B). Further analysis using droplet digital PCR (ddPCR) on the family trio showed that the CNV in the proband exhibited a complex pattern, classified as Triplication-Quadruplication (TRP-QUAD). In contrast, the father’s copy number was only marginally higher than the normal diploid level, aligning with the results obtained from aCGH (Fig. 2C). These findings highlight the intricate genetic landscape underlying the proband’s condition and confirms the presence of genetic mosaicism in the father.

To pinpoint the breakpoint junction sequences within the complex genomic structure, we conducted sr-GS on the trio. To facilitate our analysis of the sr-GS data, we employed VizCNV, a visualization tool for read depth data (https://github.com/BCM-Lupskilab/VizCNV) and phased genome data^15^. This approach revealed that the overall genomic configuration consists of a sequence of duplication-triplication-quadruplication (DUP-TRP-QUAD) (~ 448kb) (Fig. 3A). VizCNV also provided inference for approximate locations for each breakpoint. However, the genomic region spanning (chr17:37900000–38300000) was not well covered due to overlapping segmental duplications (Supplementary Fig. 1).

To ensure the accuracy of the breakpoint junction sequences, verify that no breakpoints were missed by sr-GS, and improve coverage in areas with segmental duplications, we expanded our analysis to include lr-GS for the trio. Long-read genome sequencing allowed identification of four breakpoint junctions (J1–J4) within the rearranged 17q12 locus, providing base-pair resolution of the structural variant. Two reciprocal breakpoint pairs were observed, each exhibiting opposing orientation (Fig. 3B).

Junctions J1 and J2 connect the proximal and distal ends of a ~ 4.2kb inverted segment showing an inverted duplication. Two base-pairs of microhomology were detected at J1.

Junctions J3 and J4 form a second reciprocal pair. These junctions connect the distal boundary of the triplicated region to a distal locus within the quadruplicated segment. One base-pair of microhomology was observed at J3 (Table 1).

Integration of breakpoint data with copy-number profiling supports a DUP–TRP–QUAD architecture across the locus, with two copies of the proximal segment, three copies of the intermediate segment, and four copies of the distal segment. The rearrangement encompasses multiple genes, including *DUSP14*, *SYNRG*, *DDX52*, and *HNF1B*.

No discrete breakpoint junctions were identified within the triplicated segment, despite clear copy-number gain.

To explore the CGR and the proposed structure of the SV haplotype on chromosome 17q12, we performed OGM on the proband DNA. Following the identification and sequence alignment of the breakpoint junctions, we analyzed the OGM data at these specific nucleotide positions. Although the CNVs were not captured on a single DNA molecule, *de novo* assembly of the patient’s personal genome facilitated the creation of consensus contigs that spanned the regions immediately upstream and downstream of each breakpoint. The orientation and connectivity of each junction, as initially identified in the GS data, were corroborated in the OGM. This was achieved by visualizing the directionality or polarity of sequence motifs, determining whether they were part of an inverted or direct recombinant join-point. This process is illustrated in Fig. 4. Single DNA molecules spanning the breakpoint junctions were inspected and analyzed to map and position each genomic fragment. This was done by ensuring that the visualized optical sequence motifs aligned consistently with the genomic fragment connections previously established by sequencing data.

Despite integration of long-read sequencing and OGM, the distal boundary of the rearrangement could not be fully resolved. Inspection of the reference locus revealed extensive segmental duplications in this region, which likely limit unique alignment and prevent complete delineation of the terminal structure.

## Discussion

The clinical and genetic investigation of BAB10751, a 7-year-old boy with a complex constellation of phenotypic anomalies, underscores the intricate interplay between genomic architecture and disease manifestation. The proband’s clinical presentation, characterized by intellectual disability and growth retardation, immune deficiency, multiple renal issues including nephrolithiasis, renal cysts, secondary renal failure, and distinct facial features, mirrors the complexities often found in cases of severe NDDs. The identification of a CGR involving the DUP-TRP-QUAD structure within the 17q12 region further delineates the genetic complexities that may contribute to such multifaceted clinical outcomes. Notably, the layered complexity of this rearrangement only emerged through iterative application of complementary technologies, with each successive method revealing structural features that the preceding approach had missed. Exome sequencing with read-depth analysis (HMZDupFinder) first flagged a duplication, but array CGH revealed this was in fact a more complex DUP-TRP event. ddPCR subsequently uncovered a further layer of complexity — a TRP-QUAD pattern — that was not evident by array alone, and simultaneously exposed paternal mosaicism that had been missed by aCGH. Short-read genome sequencing then refined this into a DUP-TRP-QUAD configuration and approximated breakpoint locations, while long-read sequencing resolved four discrete breakpoint junctions at base-pair resolution, including microhomology signatures suggestive of the underlying rearrangement mechanism. Optical genome mapping added an orthogonal layer of support for junction orientation and connectivity.

This progressive unmasking illustrates a broader principle: what initially appears to be a simple copy-number gain may, upon deeper investigation, prove to be a substantially more complex structural rearrangement. This case highlights the necessity of layered technological strategies in cases where conventional diagnostic methods fail to yield medically actionable information.

Notably, the genomic rearrangement spans critical genes including *DUSP14, SYNRG, DDX52*, and *HNF1B*, which have been implicated in immune, developmental, and renal abnormalities, thereby providing a plausible link to the proband’s symptoms^16–19^. These genes are likely affected through distinct mechanisms depending on their position relative to the rearrangement. *SYNRG* and *HNF1B* fall within regions of copy-number gain but are not directly disrupted by a breakpoint junction, consistent with a dosage-sensitive mechanism. In contrast, breakpoint junctions were identified directly within *DUSP14* and *DDX52*, indicating that these genes are disrupted by the rearrangement. This structural disruption may generate truncated or non-functional gene products. DUSP14 (Dual Specificity Phosphatase 14) is a member of the dual-specificity phosphatase family, which is involved in the negative regulation of cellular proliferation and differentiation by dephosphorylating both tyrosine and serine/threonine residues on target proteins^20^. This gene plays a crucial role in the regulation of immune responses and cellular signaling pathways^16^. DUSP14 is known to dephosphorylate and inactivate MAPKs (Mitogen-Activated Protein Kinases), which are essential for cellular responses to a variety of stimuli, including stress and cytokines. It negatively regulates T-cell activation by dephosphorylating key signaling molecules in the T-cell receptor signaling pathway, thereby modulating immune responses^20^. Given its role in regulating immune responses, mutations or dysregulation of *DUSP14* could lead to immune system deficiencies. The proband’s immune deficiency may be partly attributable to altered DUSP14 activity, resulting in impaired T-cell function and immune regulation. While the specific role of SYNRG in NDDs remains to be fully elucidated, its involvement in critical cellular processes including intracellular trafficking and cellular organization^21^ suggests that disruptions could contribute to the complex phenotype observed in the proband. This is further supported by a recent case series identifying biallelic *SYNRG* variants in patients with complicated hereditary spastic paraplegia (HSP) accompanied by developmental delay/intellectual disability, proposing *SYNRG* as a novel candidate gene for HSP affecting AP-1-mediated intracellular trafficking^22^. DDX52 (DEAD-Box Helicase 52) is part of the DEAD-box protein family, characterized by the conserved motif Asp-Glu-Ala-Asp (DEAD). These proteins are involved in various aspects of RNA metabolism, including RNA splicing, translation initiation, and ribosome assembly, playing crucial roles in cellular growth and division^23,24^. Since DDX52 is involved in ribosome assembly, mutations or disruptions in this gene could impair protein synthesis. This impairment could lead to developmental delay observed in the proband. Several *DDX* paralogous have been linked to NDDs including *DDX3X, DDX54*, and *DHX9*^25–27^. HNF1B (Hepatocyte Nuclear Factor 1 Beta) is an extensively studied protein and gene associated with renal development and function. Heterozygous mutations in *HNF1B* (MIM ID: 137920) are the most common monogenic causes of congenital kidney diseases, including renal cysts and diabetes syndrome (RCAD), and other renal abnormalities such as multicystic dysplasia and hypoplastic glomerulocystic kidney disease^19,28,29^. Conversely, increased *HNF1B* dosage has also been implicated in renal and structural anomalies such as annular pancreas, with functional studies in zebrafish confirming that *HNF1B* overexpression disrupts normal organ development^30^. These findings support a dosage-sensitive role for *HNF1B* in renal and developmental phenotypes, consistent with the renal cysts and secondary renal failure observed in the proband.

The minor increase in copy number observed in the proband’s father, indicative of mosaicism, adds another layer of complexity to the genetic narrative. The identified mosaicism suggests a possible germline transmission that may have gone undetected without the sensitive methodologies employed.

The intricate genomic configuration observed in the proband suggests multiple possible mechanisms. Non-Allelic Homologous Recombination (NAHR) occurs between misaligned homologous sequences, often involving segmental duplications or low-copy repeats; repetitive elements at the distal end of the CGR region could have facilitated such misalignment, contributing to the genomic instability underlying this rearrangement. Microhomology observed at several breakpoint junctions raises the possibility that a replication-based mechanism, such as microhomology-mediated break-induced replication (MMBIR), also contributed to formation of the complex rearrangement; in which multiple replicative template switches — guided by short regions of microhomology — generate the stepwise duplication-triplication-quadruplication architecture observed here.

The case of BAB10751 not only amplifies the discussion surrounding the genetic etiology of complex NDDs but also opens avenues for future research into similar cases where conventional diagnostic approaches may fall short. Further studies focused on the functional impacts of CGRs involving key developmental genes could illuminate the pathways through which such genomic alterations exert their pleiotropic effects.

## Conclusions

This case study exemplifies the critical role of advanced genomic technologies in diagnosing and understanding complex genetic disorders. It highlights the necessity of a nuanced approach to genetic analysis, which considers not only the static composition of the genome but also the dynamic and often cryptic aspects of genomic rearrangement and mosaicism.

## Supplementary Material

Supplementary Files

This is a list of supplementary files associated with this preprint. Click to download.

- SupplementaryFigure1.pptx

## Figures and Tables

**Figure 1:**
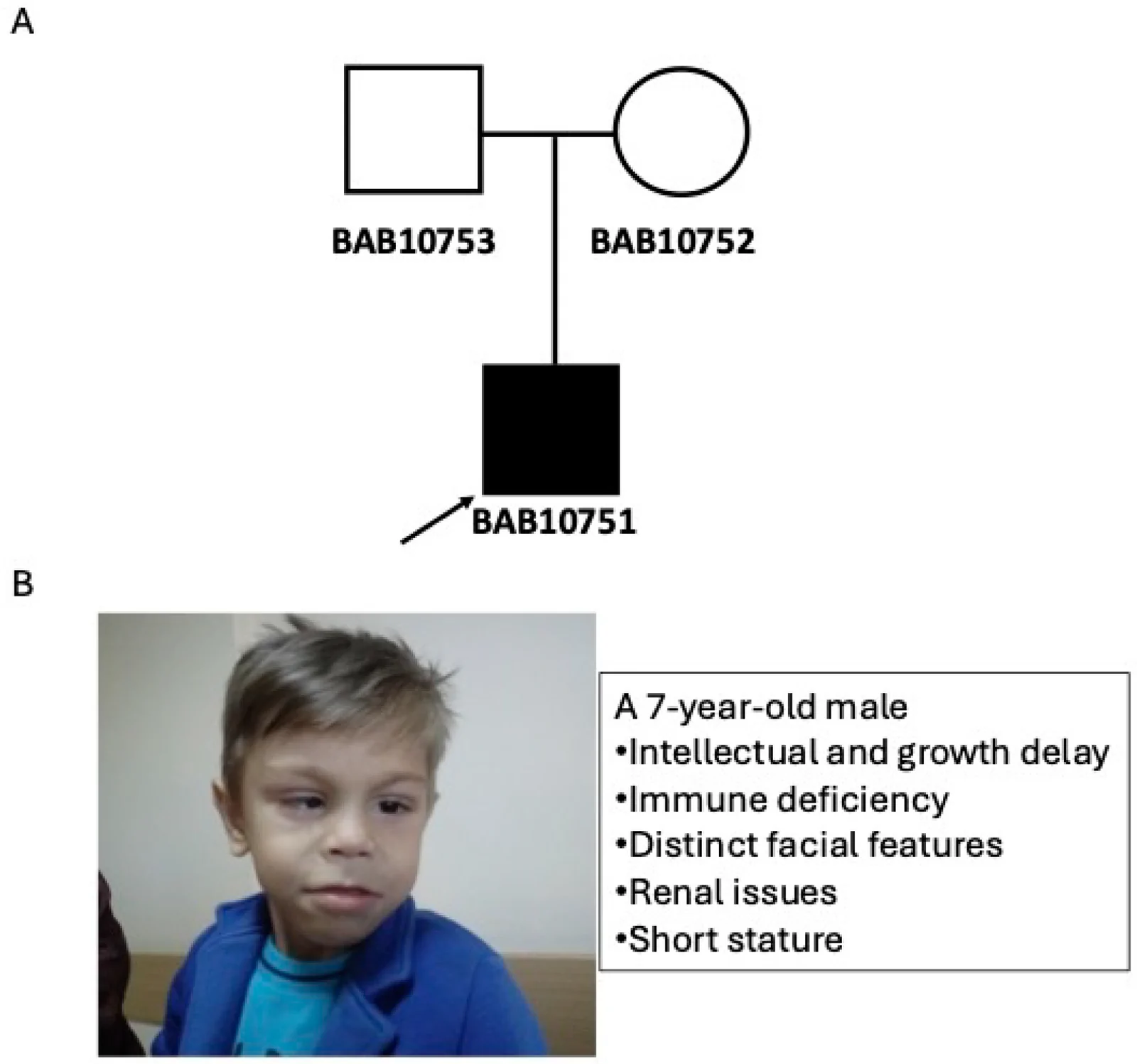
Proband BAB10751. A) family pedigree b) Images showing the distinct facial features including hypotelorism, upslanting palpebral fissures, a flat depressed nasal bridge, and a wide prominent philtrum.

**Figure 2:**
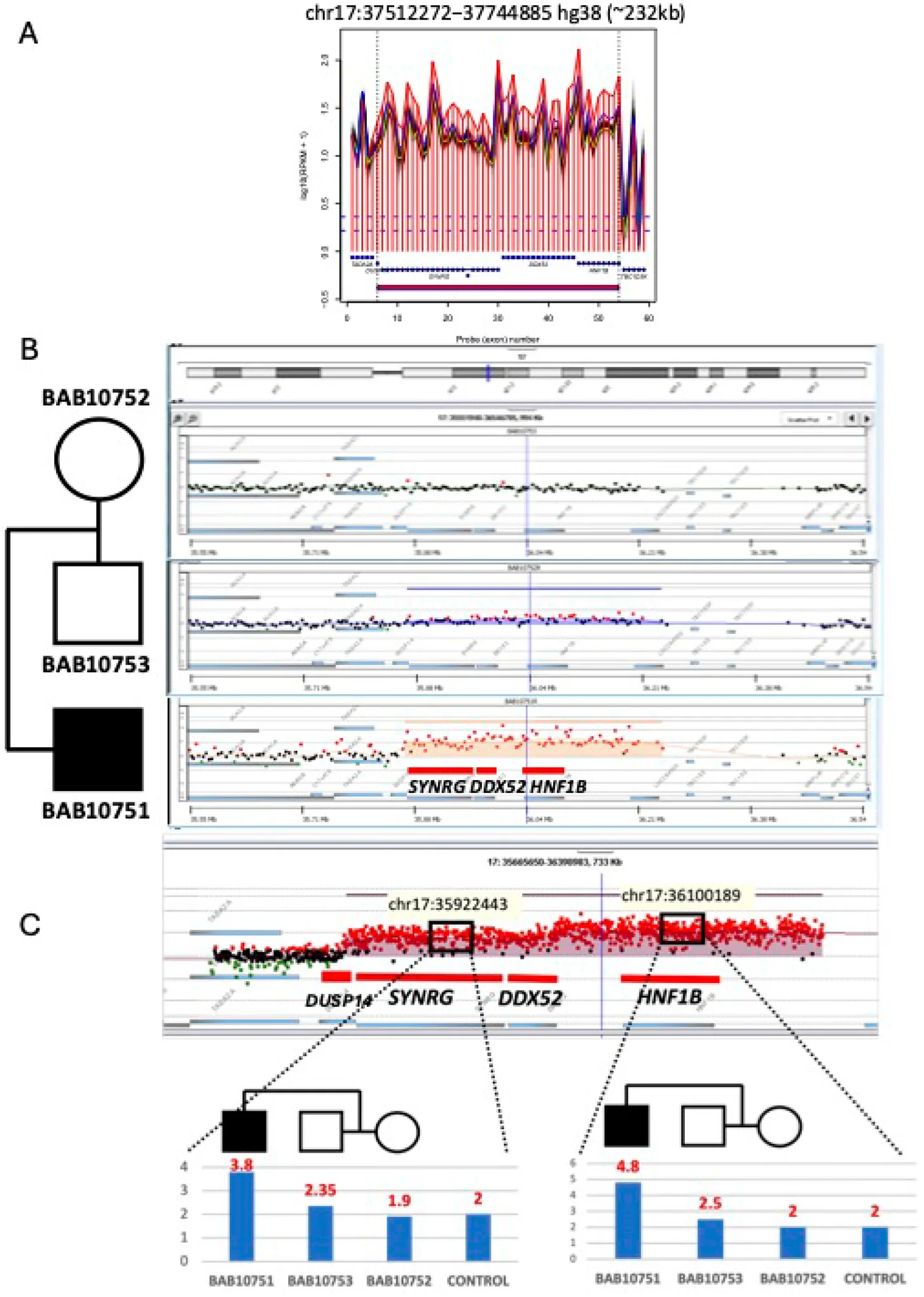
A) HMZDupFinder detected a duplication using normalized exome read count data plotted for BAB10751 with red line versus nearest samples in our database with black lines. The predicted CNV gain is ~232 kb in size spanning *DUSP14, SYNRG, DDX52*, and *HNF1B*. B) Confirmation of heterozygous CNV in the proband by aCGH showed a 307 kb “apparent” duplication-triplication CNV, where the exact position for the end of the CNV was not covered by the chr17 array due to the highly repetitive region. Father (BAB10753) showed a slight increase in copy number status suggesting mosaicism in this region. **C)** ddPCR for the trio revealed that the CNV is triplication-quadruplication in the proband, while the father’s copy number was slightly above normal (2n) consistent with the aCGH findings.

**Figure 3:**
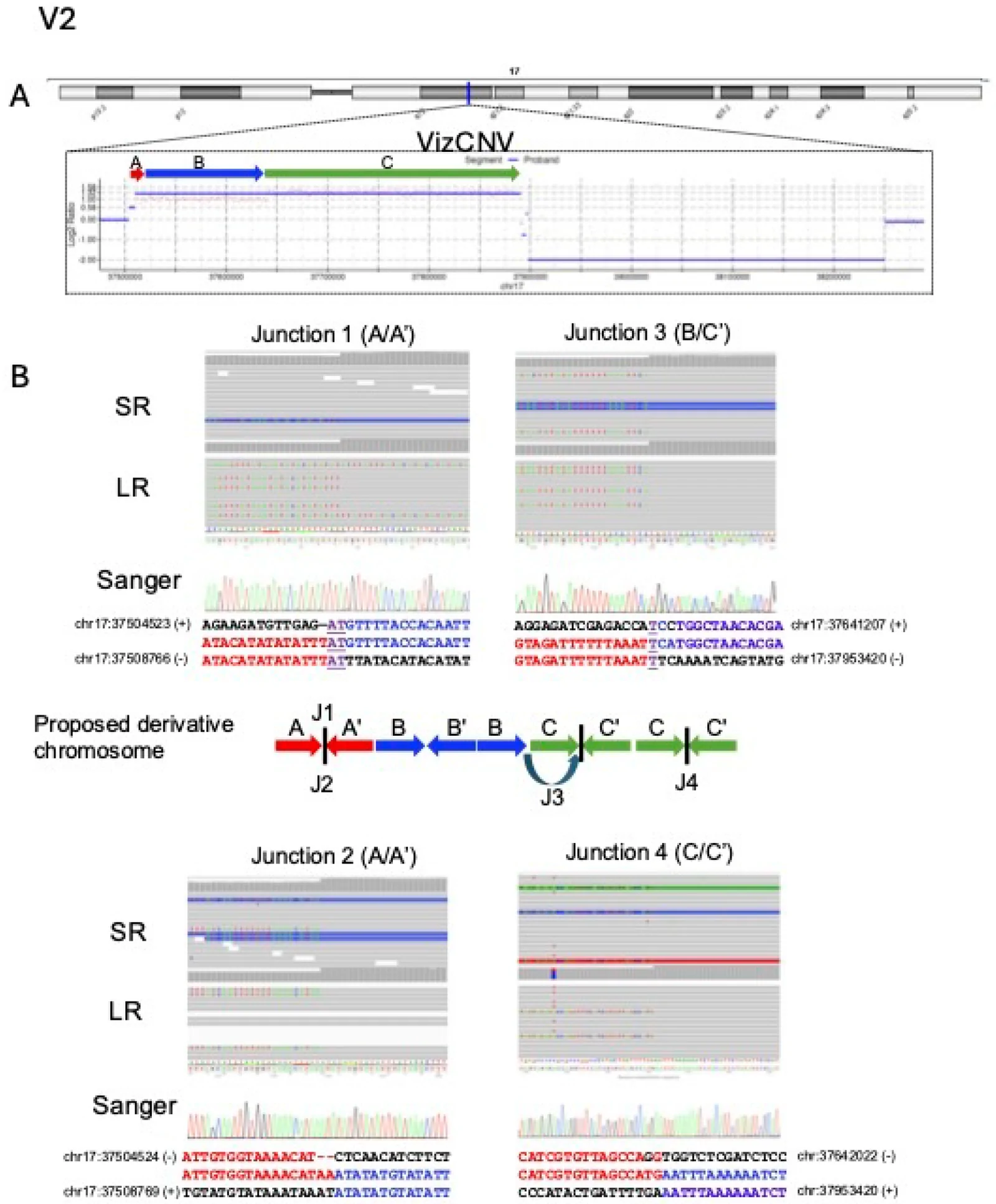
Proposed model of the derivative chromosome showing a DUP–TRP–QUAD architecture. A) VizCNV visualization of the DUP-TRP-QUAD. Dashed lines highlight a zoomed-in view of the CGR B) Breakpoint junction sequences as observed in the sr-GS and lr-GS data within the Integrative Genomics Viewer (IGV) shown as soft clipping, Sanger traces confirming all the identified junctions. The proximal segment (A) undergoes inverted duplication, followed by triplication of segment (B) without a discrete breakpoint junction, and stepwise inverted amplification of segment (C). Breakpoint junctions J1–J4 are indicated. The distal boundary likely resides within a segmental duplication region, precluding complete resolution.

**Figure 4:**
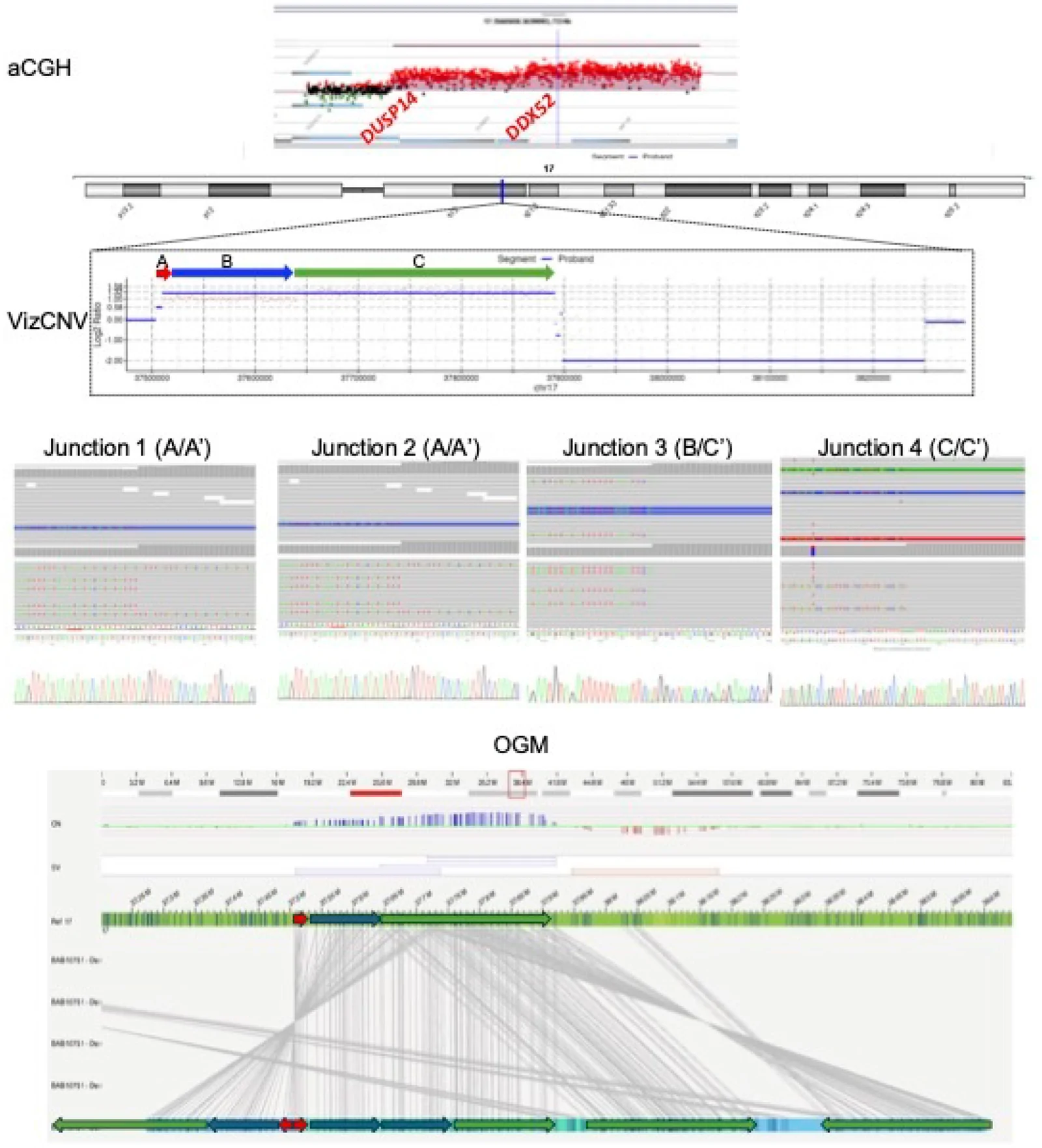
Multi-Omics Approach Resolving a DUP-TRP-QUAD Event. aCGH Analysis reveals a DUP-TRP structure at the 17q12 region, with the *DUSP14* and *DDX52* genes located at the junctions. Illumina Short-Read Genome Sequencing (SR-GS): The read depth for this region is visualized using the VizCNV plotting program. Breakpoint junction sequences detected by sr-GS, lr-GS, and Sanger sequencing. Bionano Optical Genome Mapping: This technique reveals the full architecture of the complex rearrangement. The colored arrows at the top represent the reference orientation of each genomic fragment. The arrow orientation at the bottom represents the orientation of each genomic fragment in this proband with respect to reference. All chromosomal coordinates are based on the hg38 genome assembly.

**Table 1:** Breakpoint junctions defining the 17q12 complex genomic rearrangement

Junction	Breakpoint 1 (GRCh38)	Breakpoint 2 (GRCh38)	Junction feature	Structural context
**J1**	chr17:37,504,523 (+)	chr17:37,508,766 (−)	2-bp microhomology	Reciprocal junction defining the proximal inverted duplication
**J2**	chr17:37,504,524 (−)	chr17:37,508,769 (+)	2-bp insertion	Reciprocal partner of J1
**J3**	chr17:37,641,207 (+)	chr17:37,953,420 (−)	1-bp microhomology	Junction linking the triplicated region to the distal amplified segment
**J4**	chr17:37,642,022 (−)	chr17:37,953,420 (+)	Blunt junction	Reciprocal partner of J3

## Data Availability

Exome and genome sequencing data for this family are available under controlled access through the GREGoR Consortium dbGaP study phs003047 via the NHGRI AnVIL.

## Abbreviations

aCGH: Array Comparative Genomic Hybridization
BAM: Binary Alignment Map
BCR: Balanced Chromosomal Rearrangement
BCL: Binary Base Call
BCM: Baylor College of Medicine
CGR: Complex Genomic Rearrangement
CMA: Chromosomal Microarray
CNV: Copy Number Variant
ddPCR: Droplet Digital PCR
DLE 1: Direct Labeling Enzyme 1
ES: Exome Sequencing
FISH: Fluorescence *In Situ* Hybridization
GS: Genome Sequencing
GREGoR: Genomic Research Elucidates the Genetics of Rare disease (program)
HGSC: Human Genome Sequencing Center
IGV: Integrative Genomics Viewer
IRB: Institutional Review Board
lr GS: Long–Read Genome Sequencing
MMBIR: Microhomology–Mediated Break–Induced Replication
NAHR: Non–Allelic Homologous Recombination
NDD: Neurodevelopmental Disorder
NGS: Next–Generation Sequencing
NHLBI: National Heart, Lung, and Blood Institute
NINDS: National Institute of Neurological Disorders and Stroke
OGM: Optical Genome Mapping
PFGE: Pulsed–Field Gel Electrophoresis
PMSF: Phenylmethylsulfonyl Fluoride
QC: Quality Control
RCAD: Renal Cysts and Diabetes syndrome
RPKM: Reads Per Kilobase per Million mapped reads
sr GS: Short–Read Genome Sequencing
SNV: Single–Nucleotide Variant
SV: Structural Variant
UHMW: Ultra–High Molecular Weight
